# Effect of starvation on electrotaxis response

**DOI:** 10.17912/micropub.biology.000962

**Published:** 2023-09-15

**Authors:** Shane K. B. Taylor, Muhammad H. Minhas, Bhagwati P. Gupta

**Affiliations:** 1 Biology, McMaster University, Hamilton, Ontario, Canada

## Abstract

*Caenorhabditis elegans* is an ideal model for investigating the effects of extrinsic and intrinsic conditions on the behavioral changes of animals. Our group previously showed how different conditions influence the behavior of worms following an electric stimulus in a microfluidic channel, known as electrotaxis. In this study we describe the effect of starvation on the electrotaxis movement of animals. We show that acute starvation did not affect the electrotaxis response or dopaminergic neurons but extended the lifespan of animals.

**Figure 1. Effect of different starvation models on electrotaxis:**
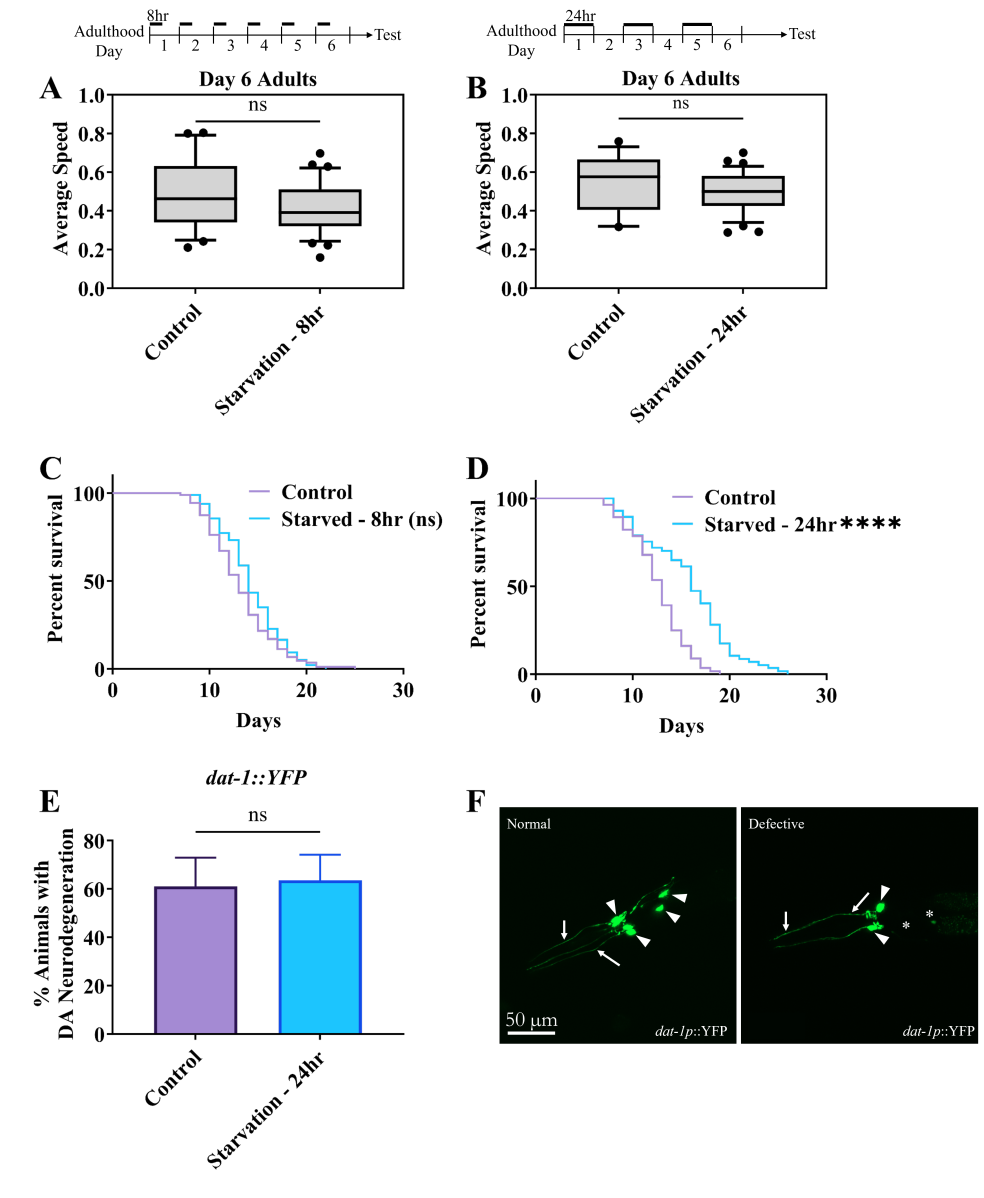
**(A, B)** Effect of starvation on electrotaxis speed. Boxes represent measurements from 25th to 75th percentiles, central horizontal lines represent medians, vertical lines extend to 10th and 90th percentiles, and dots represent outliers. **(A)** Animals are starved from day 1 adulthood everyday for 8hrs until day 6 of adulthood. **(B)** Animals are starved every alternate day for 24hrs starting from day 1 until day 6 of adulthood. In **A and B** there was no significant difference in electrotaxis behaviour following starvation diets ( **A,** p = 0.0592 & **B** , p = 0.1102). **(C, D)** Lifespan analysis began from day 6 of adulthood. **(C)** Lifespan analysis of animals starved from day 1 adulthood everyday for 8hrs until day 6 of adulthood. Mean lifespan of starved worms (mean 14.216 ± 0.318) remained unchanged compared to fed control (mean 13.205 ± 0.364) (p = 0.097). ( **D)** Lifespan analysis of animals starved every alternate day for 24hrs starting from day 1 until day 6 of adulthood. Animals starved for 24hrs had an increased mean lifespan (mean 15.754 ± 0.607) (p <0.001) compared to fed controls (12.625 ± 0.383). **(E)** Quantification of neuronal degeneration of dopaminergic (DA) neurons in day-6 adults which followed the same starvation protocol as **(B and D).** There was no significant change in DA neurodegeneration compared to untreated controls (p = 0.8737). **(F)** Representative images of animals showing normal and defective DA neuron morphology using *dat-1p::* YFP marker. Arrows point to dendrites and arrowheads mark cell bodies. The stars in the defective animal mark missing or faint cell bodies and axons. Note that in the defective worm dendrites are not as smooth compared to the normal animal and blebs can be seen by the arrows. Normal and defective dendrites were observed in both populations. The numbers of animals were ( **A)** Control: n = 29, Starved -8hr: n = 34. ( **B)** Control: n = 17, Starved -24hr: n = 39. ( **C)** Control: n = 88, Starved -8hr: n = 97. ( **D)** Control: n = 56, Starved -24hr: n = 57. ( **E)** Control: n = 18, Starved -24hr: n = 22. **A** , **B** and **E** were analyzed using an unpaired Student’s t-test, **C** and **D** were analyzed using the log-rank (Kaplan-Meier) method for lifespan curves.

## Description

Extrinsic conditions have the capability of impacting the health of organisms. To overcome this impact, animals have evolved mechanisms to protect and mitigate these harmful conditions (Higuchi-Sanabria et al. 2018; Dutta et al. 2022) . Some of these protective responses involve the activation of signaling cascades collectively known as the stress response. The stress response works to maintain cellular homeostasis within the cell and prevent cell death (Taylor and Hetz 2020) . However, genetic (e.g., mutations) or external factors such as starvation can impair the stress response, leaving the cell vulnerable to harmful conditions. In contrast, mild stress has been shown to have beneficial effects on organisms. This is due to a process known as hormesis, whereby mild stress can improve the tolerance of organisms towards additional stressors (Shore and Ruvkun 2013; Matai et al. 2019) . For example reduced food intake (i.e., dietary restriction) has been shown to improve the health of animals through hormesis (Matai et al. 2019) .

Our lab has previously shown that multiple stress response pathways contribute to the electrotaxis behaviour in *C. elegans* , which is a movement response when animals are exposed to a DC electric stimulus (Rezai et al. 2010) . Specifically, the impairment of the stress response through mutations and external conditions such as heat and exercise reduced the electrotactic movement of animals (Taylor et al. 2021) . We previously tested *eat-2* mutants which are chronically dietary restricted due to bacterial avoidance and other conditions including slower pharyngeal pumping (Bansal et al. 2015; Kumar et al. 2019; Matai et al. 2019) . Our results showed that these animals have defects in electrotaxis (Taylor et al. 2021) . However, an acute starvation treatment had no obvious effect on the electrotaxis of animals.

In this report, we describe the effect of different dietary models on worms. We utilized two different acute starvation protocols adapted from published findings (Honjoh et al. 2009) . In the first dietary model, we starved animals for 8hrs every day until day 6 of adulthood. The second model involved 24hrs starvation treatment every alternate day starting from day 1 of adulthood until day 6. The examination of the electrotaxis speed and lifespan of such treated animals (i.e. 8hr and 24hr conditions) showed no significant effect on their electrotaxis behaviour **( Fig. 1A & B)** . Additionally, no change in lifespan was observed using the 8hr treatment but as expected from published studies, the lifespan of animals subjected to 24hr starvation treatment was increased **( Fig. 1C & D)** (Honjoh et al. 2009) .

Dopaminergic (DA) neurons were shown previously by our group to mediate electrotaxis behaviour. Therefore, we examined these neurons in 24hr treated animals but saw no significant difference from the control **( Fig. 1E & F)** . Together with our published findings, these data demonstrate that acute starvation does not affect electrotaxis (Taylor et al. 2021) . Furthermore, since *eat-2* mutants show defects in electrotaxis (Taylor et al. 2021) , this suggests differences between acute and chronic dietary restriction affecting electrotaxis of animals.

## Methods

**Strain and growth conditions**

Worms were grown at 20°C on standard nematode growth media plates seeded with *E. coli* OP50 . The strains used in this study are N2 (wildtype *C. elegans* ) and DY353: *bhEx138 [pGLC72(Cel-dat-1::yfp)]*

**Starvation Protocol**

This protocol was adapted from previous literature with modifications (Honjoh et al. 2009) . In brief, two paradigms were tested, either 8 hrs starvation everyday or 24 hrs on alternate days. In the first paradigm, i.e., 8hr treatments, worms were washed with M9 buffer at least three times to get rid of residual bacteria. Animals were then placed on NGM plates containing no food and transferred back to OP50 bacteria containing plates at the end of the starvation period. The starvation treatment for the second paradigm was the same except that it was performed on alternate days for 24 hrs. The treatment was ended on day 6, at which point animals were tested.

**Lifespan analysis**

Lifespan experiments were conducted as previously described at 20°C (Mallick et al. 2020; Taylor et al. 2021) . Experiments were performed on OP50 plates seeded with *E. coli* . Synchronized animals were transferred onto plates at day 6 adult stage following their respective starvation protocols. They were observed every day throughout the rest of their lifespan.

**Electrotaxis Protocol**

The electrotaxis assay protocol has been described previously (Tong et al. 2013) . In brief, a microfluidic channel was used which is 5cm long, 300 µm wide and 80 µm deep with electrodes on both sides of the channel. A detailed description of fabricating the device was published earlier from our lab (Rezai et al. 2010; Tong et al. 2013) . Worms are then suspended with M9 into a falcon tube and introduced into the microfluidic channel using a syringe under a dissecting microscope. Worms were then subjected to an electric field of 3 V/cm, which introduces a swimming response causing the worms to travel from anode to cathode resulting in electrotaxis. The electrotaxis response is recorded and locomotory data is extracted from videos using a MATLAB- based worm tracking software. The electrotaxis speed of animals is plotted as box plots.

**Dopaminergic neuron analysis**

Scoring of dopaminergic neurons (DA) was done using a previously published protocol (Taylor et al. 2021) . In brief the number of DA cell bodies were counted, and dendritic morphology was observed under a Nomarski fluorescence microscope. Animals with reduced cell bodies, and abnormal dendrites with blebbing, punctate pattern, deformed shape, faint appearance or complete absence were counted as defective. Wildtype animals have three pairs of DA neurons and smooth dendritic projections in the head region.

**Statistical analysis**

For lifespan analysis, all statistics were performed using SigmaPlot software 14. Survival curves were estimated using the Kaplan-Meier test, and differences among groups were assessed using the log-rank test. Survival data are expressed relative to the control group. Other statistics were performed using Graphpad Prism 9.5.1
